# GEODESIC LENGTH MORPHOMETRY: A simple method for quantifying severe degeneration laterally in rodent models of retinal photic injury

**DOI:** 10.1016/j.mex.2026.104183

**Published:** 2026-09-29

**Authors:** Kentaro Ohishi, Masafumi Ohtsubo, Akira Obana, Yoshihiro Hotta, Yu Nagashima, Shinsei Minoshima

**Affiliations:** a Institute of Photonics Medicine, Hamamatsu University School of Medicine, 1-20-1 Handayama, Chuo-ku, Hamamatsu, 431-3192, Japan; b Department of Ophthalmology, Seirei Hamamatsu General Hospital, 2-12-12 Sumiyoshi, Chuo-ku, Hamamatsu, 430-8558, Japan; c Department of Ophthalmology, Hamamatsu University School of Medicine, 1-20-1 Handayama, Chuo-ku, Hamamatsu, 431-3192, Japan; d Organization for Innovation and Social Collaboration, Shizuoka University, 3-5-1 Johoku, Chuo-ku, Hamamatsu, 432-8561, Japan

**Keywords:** Retinal light damage, Light-induced retinal degeneration, Geodesic length morphometry, Geodesic index, Geodesic equidistant-projection plot

## Abstract

Quantification of degeneration extent in curvilinear microscopic specimens, such as the retina, is challenging due to inherent geometric complexity. In the rodent retinal photic injury model, degeneration arc lengths are a critical, yet difficult-to-quantify parameter for assessing severity. We developed a novel, precise platform, termed “*Geodesic Length Morphometry*,” that quantifies the actual arc lengths of curvilinear structures using a precisely determined digital scale factor. In this methodology, defining a curved coordinate axis along the retinal contour enables precise numerical localization of any position and the measurement of dorsoventral arc lengths between any two points. This robust platform resulted in two analytical innovations. One is a new visualization method, termed “*Geodesic Equidistant-Projection Plot*,” which converts arc length data measured from curved anatomical imagery into a cumulative line chart, allowing for the direct visual comparison of actual degeneration arc length and location. The second is the definition of the “*Geodesic Index*” (0 ≤ *x* ≤ 1), which normalizes the degeneration arc length against the entire retinal arc length to enable an objective, size-independent comparison of extent of degeneration. The Geodesic Length Morphometry platform offers a robust standard for the quantitative analysis of curvilinear tissue arc length and serves as a versatile tool applicable to diverse biological and morphometric fields.

## Graphical abstract

**Figure:**
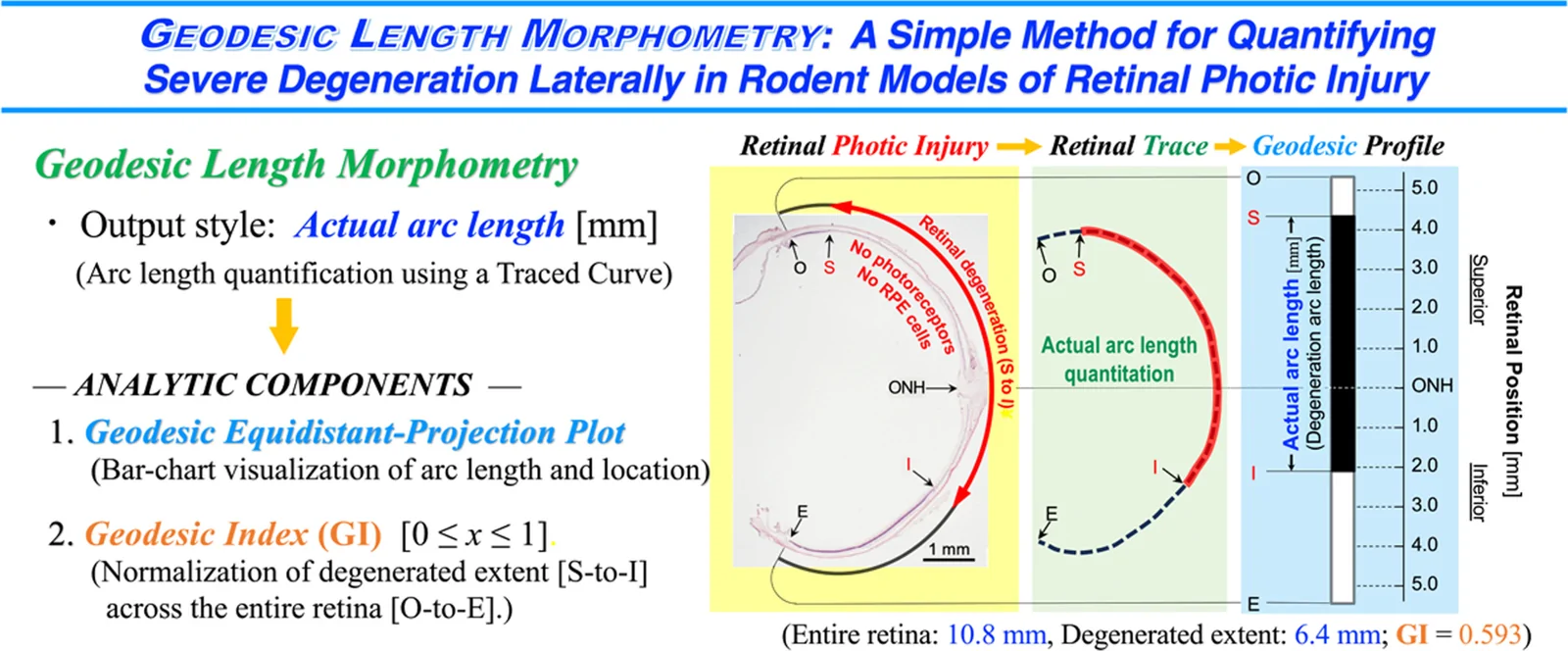

**Specifications table.**

**Table:** 

**Subject area**	Neuroscience
**More specific subject area**	*Ocular pathology in rodents*
**Name of your method**	Geodesic Length Morphometry (with Geodesic Equidistant-Projection Plot & Geodesic Index)
**Name and reference of original method**	Not applicable.
**Resource availability**	Not applicable*.*

## Background

Exposure to bright light in animals results in selective degeneration of the outer retina, known as the *Retinal Photic Injury* (*RPI*) model. This degeneration is characterized in histological ocular specimens by the thinning or disappearance of the outer nuclear layer (ONL) [1]. Historically, various unique morphometric approaches have been used to describe this morphological feature, and many of these approaches have focused on the ONL to evaluate degeneration.

Ophthalmic researchers have previously designed and improved methods to evaluate retinal degeneration. In 1970, O’Steen provided retinal thickness data at 520 µm from the optic nerve head (ONH) for female rats subjected to 4–30 days of constant light exposure [2]. In 1974, O’Steen et al. reported age-dependent light-induced photoreceptor degeneration in albino rats, wherein ONL and retinal thicknesses in six different retinal locations were measured with an interval of 1 mm along the entire vertical meridian and averaged them, thus providing a single parameter per eye [3]. In 1977, O’Steen and Kraeer published a line chart showing ONL thickness in 12 locations [4]. In 1980, Rapp and Williams developed this data into a well-known schematic representation of photic injury distribution along the vertical meridian of the rat retina [5]. This graphical representation has since become the standard framework for illustrating RPI pathology in rodents. This method provides a visualization of RPI severity in rodent eyes by measuring nuclear counts and the thickness of the ONL, photoreceptor layer, and entire retina in the sagittal sections.

Similarly, there have been reports on the length of the entire retina or the degenerated retina. In 1996, Fan *et al*. reported that the length of retinal degeneration from the superior ora serrata can be measured using light microscopy with a scope reticle [6]. However, the length measured by this method was limited to 2 mm at most. In 2016, Hendrickson reported that the retinal lengths from nasal to temporal retina in fetal and postnatal human eyes were measured along the horizontal meridian using a micrometer caliper [7]. However, this method is considered somewhat inaccurate in tracing the precise length of the curved retina, as a linear caliper provides only a series of chord-like approximations. Polosa *et al*. illustrated the RPI-affected superior/inferior rat retina by linearly reconstructing 12–13 consecutively connected histological micrographs [8]. Their pioneering work successfully demonstrated the value of quantifying the lateral extent of the injury. However, an easy-to-implement framework for standardized lateral metric analysis is still lacking in the literature.

Based on our previous rat pathology observations of rat pathological specimens [1], we contend that the degeneration arc length is a critical indicator of RPI severity. However, conventional morphometric methods inherently fail to account for the arc length within the curvilinear retina. In this paper, we introduce “*Geodesic Length Morphometry*,” a novel method designed to precisely quantify degeneration arc length in rodent specimens. By establishing a normalized scale, our approach provides a reliable, objective metric for comprehensive assessment and visualization of RPI pathology.

## Method details

### Reagents

Mayer’s hematoxylin and eosin Y solutions were purchased from Muto Pure Chemicals Inc. (Tokyo, Japan). All other reagents were obtained from FUJIFILM Wako Pure Chemical Co., Ltd. (Osaka, Japan).

### Definition of geodesic length morphometry

To overcome the limitations of conventional linear measurements, we developed a novel morphometric approach, “*Geodesic Length Morphometry*,” that enables precise quantification of the arc length along the curved retinal contour. By tracing the true arc length of histological sections, this method allows for the accurate mapping and visualization of lesion distributions through standardized retinal charts, named “*Geodesic Equidistant-Projection Plot*.” Furthermore, to facilitate objective comparisons across samples with varying eye sizes, we established a normalized scale, where the “*Geodesic Index”* (“GI”; 0 ≤ *x* ≤ 1) represents the relative degeneration extent within the entire retina. This integrated framework provides a consistent lateral metric for both the detailed assessment and the visual representation of RPI pathology.

### Animals and housing conditions

A total of 10 male (for method validation) and 2 female (for pictures) WKY/Izm rats were used in this study. The rats were purchased from Japan SLC, Inc. (Hamamatsu, Japan), which supplies the strain officially sustained by the Disease Model Cooperative Research Association (Kyoto, Japan). The rats were housed in an environment with a 12-h light/dark cycle and provided with *ad libitum* access to an autoclaved standard pelleted diet and tap water.

### Bright light exposure protocol

To induce retinal degeneration, adult rats were dark-adapted for 29 h starting at 19:00, then exposed to a 3,000 lx white fluorescent light (20 W; FL20SSD/18; NEC, Tokyo, Japan) for 3 h. Following the light challenge, the animals were allowed to recover in darkness until 07:00 the next morning. During the 3-h exposure period, food and water were temporarily removed to ensure uniform light distribution to the eyes.

### Tissue processing and histological analysis

The animals were euthanized via an overdose of inhaled isoflurane. Enucleated eyes were initially fixed in 4% paraformaldehyde (0.1 M phosphate buffer, pH 7.4) for 10 min on ice. After removing the inferior half of the cornea, lens, and vitreous body under a stereomicroscope (SMZ-1; Nikon Co., Tokyo, Japan), the resulting eyecups were further fixed at 4 °C for 4 h. Following cryoprotection and embedding, 7-µm-thick sagittal sections were prepared using a cryostat (CM 1510-11; Leica Microsystems GmbH, Wetzlar, Germany). The sections were then stained with Mayer’s hematoxylin and eosin Y, dehydrated through a graded ethanol series, cleared in xylene, and mounted under coverslips. Histological evaluation was conducted using a light microscope (BX-50; Olympus Co., Tokyo, Japan) equipped with a digital camera (Pro 600ES; Pixera Co., Los Gatos, CA).

### Determination of reference dash pitch

The arc length of structures in the micrographs was determined using Microsoft® PowerPoint® v2008 or later (*see* Supplemental material). A dashed curve was drawn along the curved retinal tissue using the software’s built-in drawing function, and dash counts from the zero point were recorded to quantify the length. Consequently, a digital scale factor was required to convert the dash count to the actual arc length. Therefore, a reference dash pitch, *P,* was determined by comparing dashed lines with the micrograph of a 10-mm stage micrometer glass slide, which was graduated into 100 divisions with a pitch of 0.1 mm (Fig. 1).

**Fig. 1:**
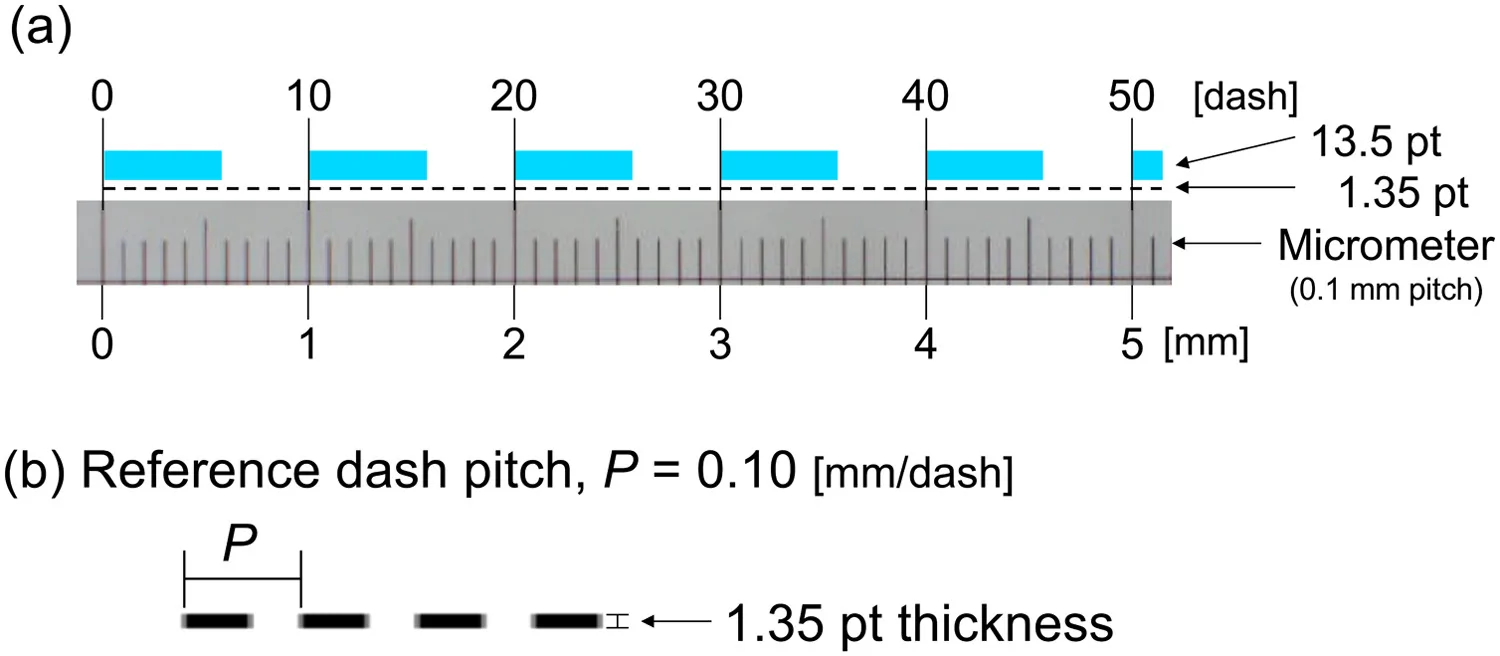
Determination of reference dash pitch. (a) Calibration setup using a stage micrometer: Thin (1.35 pt) and thick (13.5 pt) dashed lines were drawn adjacent to stage micrometer micrograph (0.1 mm/division). Dash count ratio between thin and thick dashed lines is 10:1. (b) Derivation of reference dash pitch: Reference dash pitch, *P*, is 0.10 mm/dash. This value is derived from accurate correspondence between 50^th^-dash position on 1.35-pt line and 50^th^-division on the stage micrometer in panel (a).

For this purpose, a micrometer micrograph was captured at 2× objective lens magnification (Fig. 1a), and subsequently loaded into Microsoft PowerPoint (Microsoft Japan Co. Ltd., Tokyo, Japan). The picture file was dragged and dropped onto the slide (picture size was 21.59 cm × 16.21 cm), although the picture size may depend on the microscope/camera system used. Following this, thin (1.35 pt) and thick (13.5 pt) dashed straight lines were drawn next to the micrometer scale (Fig. 1a). The thick line was used for easier counting of the dash count in the thin line, resulting in a 10:1 dash count ratio between the thin and thick lines. Finally, the *P* value was calculated to be 0.10 mm/dash by comparing the thin dashed line and the micrometer picture (Fig. 1b).

### Geodesic Length Morphometry

The size and location of the retinal degeneration, which had completely lost its photoreceptors and retinal pigment epithelial cells, were measured in the sagittal plane of the stained rat eye section. In brief, a micrograph of the eye with the ONH on the underside was taken under 2× objective lens magnification and loaded onto a PowerPoint slide.

The specific morphometric points in the eye micrograph (Fig. 2a) were marked, using higher-magnification micrographs (10× objective lens) (Figs. 2b and 2c). The five determined positions were designated as follows: the superior ora serrata was designated as O; the superior and inferior edges of the retinal degeneration were designated as S and I, respectively; the ONH was designated as H; and the inferior ora serrata was designated as E (Fig. 2).

**Fig. 2:**
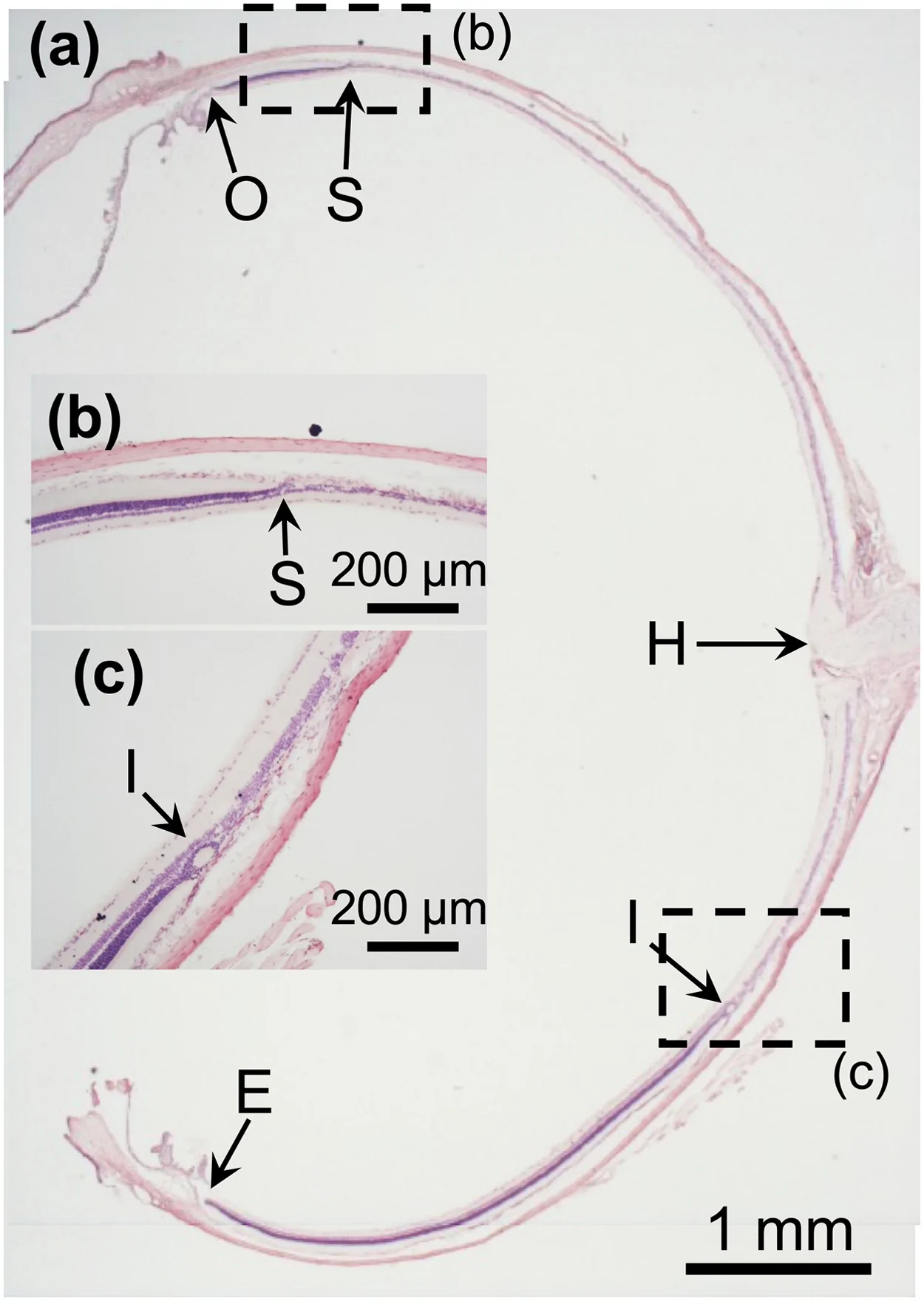
Location of each morphometric point. (a) Pathological micrograph of a sagittal eye specimen showing retinal degeneration at 2× objective magnification. (b, c) Higher-magnification images (10× objective) highlighting superior and inferior edges of patchy retinal degeneration. Guided by cellular-level details in panels (b) and (c), specific morphometric landmarks—O, S, H, I, and E—were identified and mapped onto lower-magnification view panel (a).

The contours of the outer retina were traced using the “Curve” tool in Microsoft PowerPoint. The coordinate data manually plotted using the tool are interpolated as a continuous path consisting of spline segments generated according to the Catmull–Rom algorithm [9]. The positional data for the first and second control points of these generated splines were transferred, without modification, to the “cubicBezTo” elements within the internal eXtensible Markup Language (XML) data of the file, where they were represented as cubic Bézier curves. The involvement of the Catmull-Rom algorithm was verified by extracting the coordinate data from the XML and confirming that the values matched those calculated by substituting the points into the theoretical equations for the first and second control points. Their correspondence was observed for all segments except for those at both ends; at these terminal segments, the mathematical requirements for the Catmull–Rom algorithm were not met, suggesting that one or more algorithms may be combined to define it (*see* Supplementary material).

For precise quantification, the curve was formatted as a 1.35 pt dashed line. To facilitate accurate counting of the dashes, this curve was duplicated and thickened to 13.5 pt using a different color; by superimposing this thickened curve beneath the original curve, each segment of the thick dashed line served as a visual marker for every 10 dashes of the thinner line (Fig. 3). The dash count ratio between the thin and thick lines was 10:1. The dashes in the thin curve were counted clockwise from O to the four other positions (S, H, I, and E) (Fig. 3). These results were assigned to variables of *S, H, I*, and *E* (in *italics*), respectively.

**Fig. 3:**
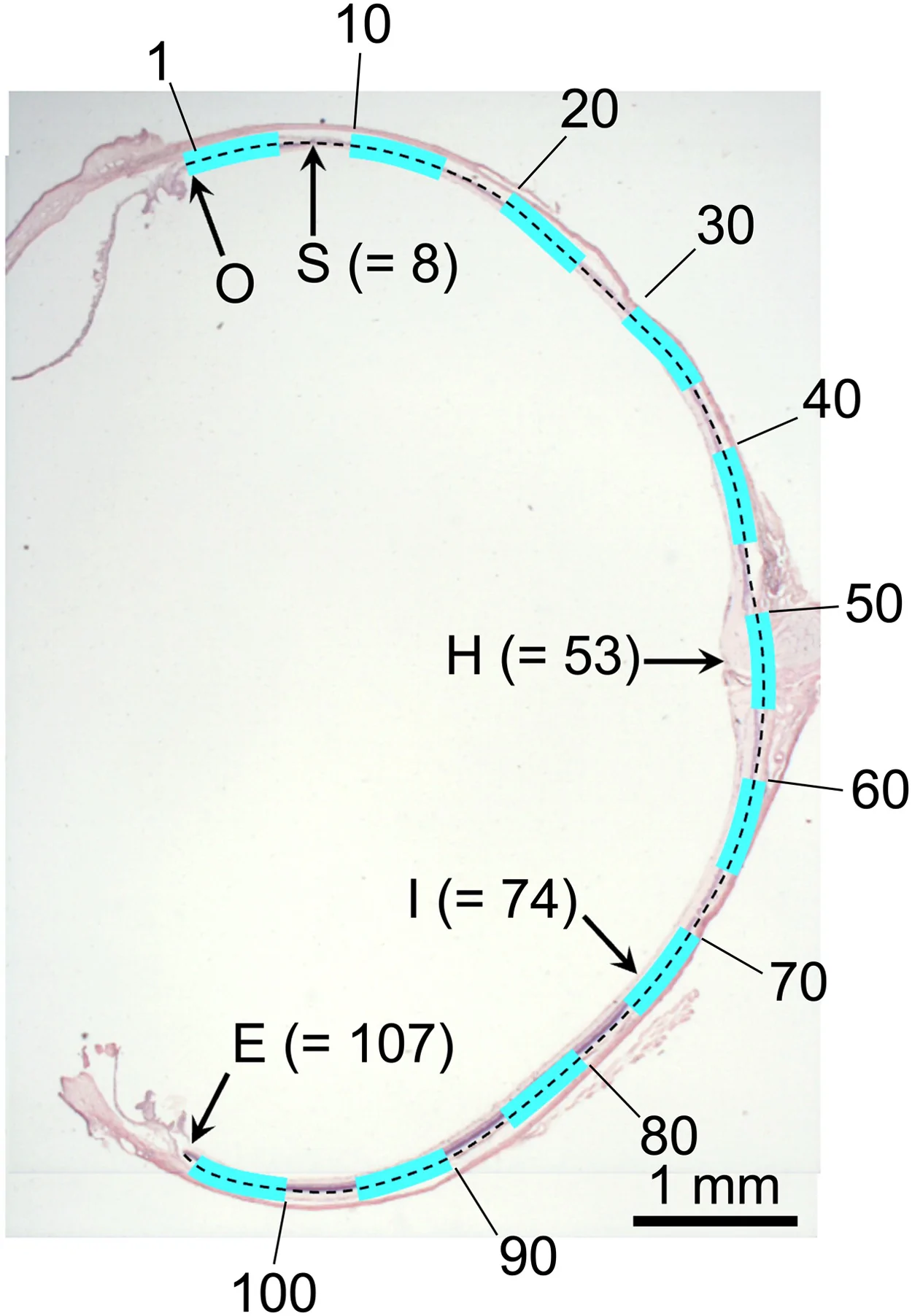
Preparation of dashed curves for the quantification of positional parameters. Thin dashed line (1.35 pt) was traced along outer retina from O to E. To facilitate accurate quantification, this curve was duplicated, and weight of the duplicate was increased to 13.5 pt and changed to light blue for enhanced contrast. This thicker line was positioned beneath the original dashed line to serve as a visual guide. Numerical values outside the globe indicate the counts of dashes measured clockwise from O to each designated position.

The measured arc length was calculated as the product of the dash count and the reference dash pitch (*P*) [Eqs. (1) and (2)]. The ratio of the degeneration arc length to the entire retinal arc length was calculated using Eq. (3), which defined “*Geodesic Index*” (GI; 0 ≤ *x* ≤ 1). The retinal degeneration arc length was calculated by multiplying the GI and the entire retinal arc length [Eqs. (1) and (3)].

(1) Entireretinalarclength[mm]=E·P

(2) Degenerationarclength[mm]=(I−S)·P

(3) $$\text{Geodesic}\;\text{Index}\;\left(\text{GI}\right)=\frac{I\;-\;S}{E}\;\;\text{for}\;0\leq \text{GI}\leq 1$$

Retinal degeneration was visualized across the entire retina using a four-segment stacked bar chart in Microsoft® Excel® (Microsoft Japan Co. Ltd.) (Fig. 4). The length of each segment was calculated using four dash counts along the retina and specific formulae. The resulting chart shows the arc length and location of degeneration along the entire retina. The vertical positions of all charts were aligned to the ONH position with the scaling parameter, *F*. Here, *F* was set to 6, a constant tailored to the dimensions of the rat retina. *F* enables calculation of the bottom segment, which serves as a transparent spacer for the gap created by this vertical alignment. This allowed the ONH location, H, of multiple eye samples to be aligned at the same height, *F*, to visualize the precise location of the degeneration relative to the ONH, a critical factor in determining the onset and progression of the injury, while also objectively representing its extent. This chart was termed “*Geodesic Equidistant-Projection Plot*.”

**Fig. 4:**
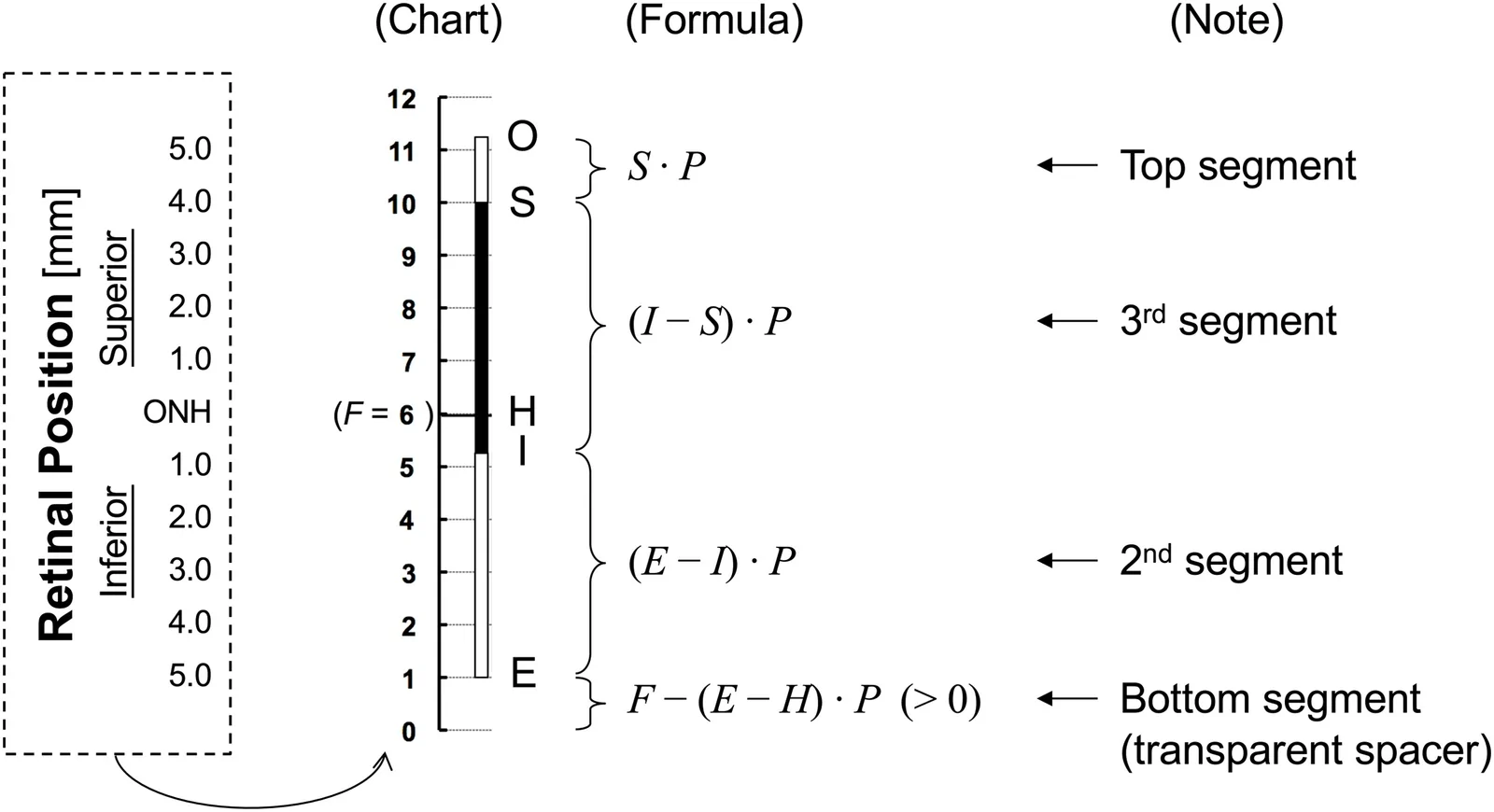
*Geodesic Equidistant-Projection Plot* of photic injury and calculation guidelines. Stacked bar chart visualizes the arc length and location of retinal degeneration across the entire retina. Plot is constructed using four segments, whose lengths are calculated based on the indicated specific formulae using reference dash pitch, *P*, and dash counts from the origin, O, to the specified anatomical position of S, H, I, and E. The chart’s key feature is its vertical alignment to ONH position, H, which standardizes visualization of degeneration location relative to the ONH and provides a comprehensive view of retina’s arc length. Alignment is achieved by defining a scaling parameter, *F* [= 6; where *F* > (*E* − *H*) ⋅ *P*], which sets the consistent height for the ONH. A bottom segment (transparent spacer) is a calculated gap required to vertically align position H to height *F*, ensuring standardized placement of the ONH location.

In addition, this method allows the identification of particular retinal positions and calculation of the arc length between them using certain parameters. The ONH is used as a standard anatomical reference for the analysis of rodent eyes, and the arc length from the ONH to particular positions in the retina is crucial information. Regarding the central position of retinal degeneration, C [*C*: the dash count from O to C, that is, *C* = (*S* + *I*) / 2], the formula for calculating the GI of the ONH to the central position (GI _H–C_) is established as shown in Fig. 5. This formula is presented as follows:

(4) $${\mathrm{GI}}_{H-C}\;=\;\frac{H\;-\;C}{E}\;=\;\frac{2H\;-\;S\;-\;I}{2E}$$

**Fig. 5:**
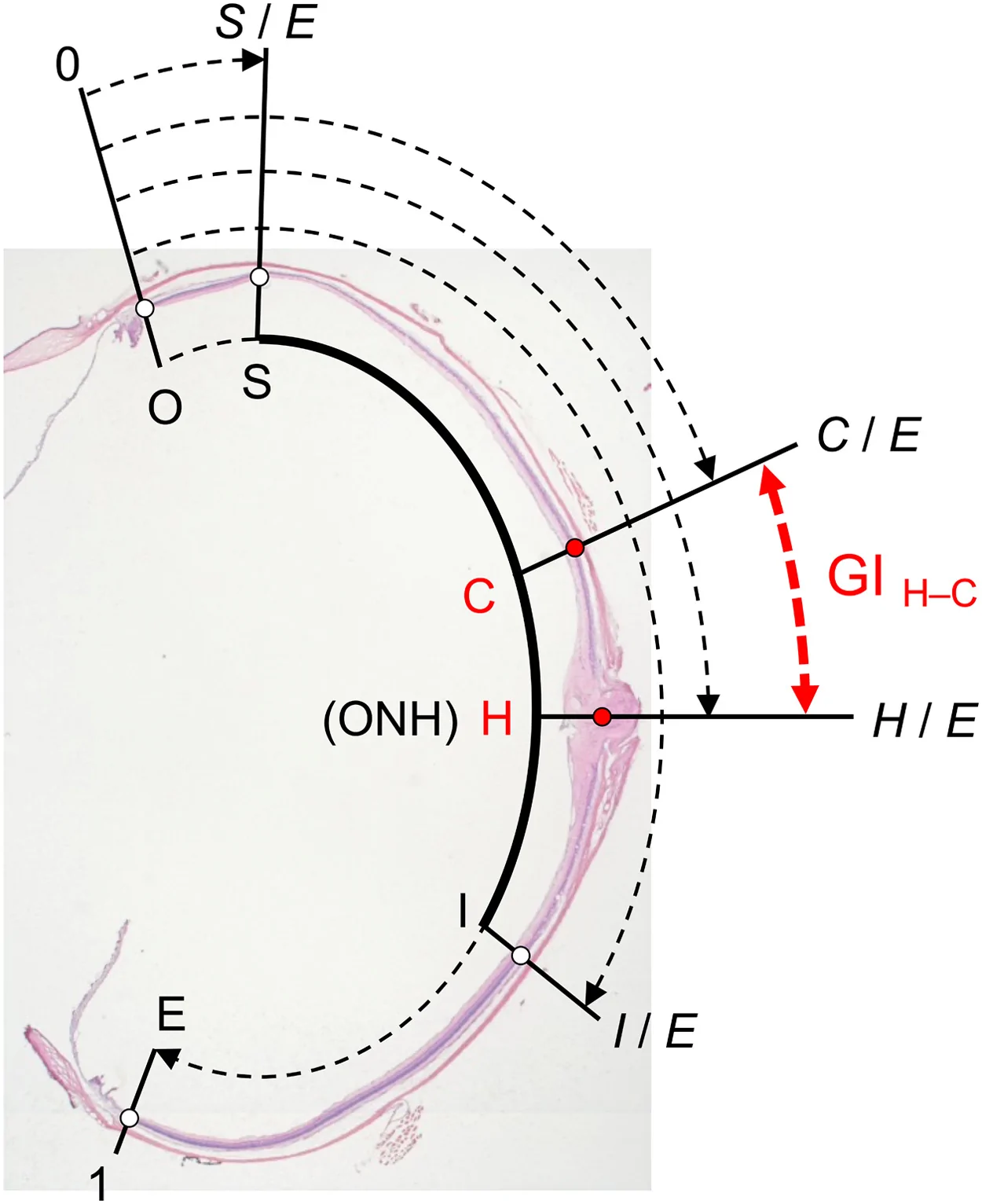
Derivation of the formula determining the GI of the arc length between the ONH and the central position of retinal degeneration. Dash count of the central position of degeneration, *C*, from O to C is defined as midpoint of S and I of the degeneration: *C* = (*S* + *I*) / 2. GI of the arc length between ONH, H, and central position of degeneration, C, is given by the formula: GI _H–C_ = (*H* – *C*) / *E*. The final formula for the GI of the arc length between H and C is: GI _H–C_ = (2*H* − *S* – *I*) / 2*E* (Eq. 4).

For the arc length between two arbitrary positions, X and Y, the general formula for calculating the GI is established as the absolute value of the difference in their normalized coordinates. This calculation extends the underlying principle of GI using the normalized arc length as a universal positional metric. The formula is presented as:

(5) $${\mathrm{GI}}_{X-Y}\;=\;\frac{X\;-\;Y}{E}$$

where *X* and *Y* are the dash counts from O to the respective positions. Thus, when comparing and analyzing multiple individuals, it is logical to use GI, which is normalized to the eye size of each individual. The comprehensive methodology developed herein is termed “*Geodesic Length Morphometry*.”

## Summary of methodology

Using the *Geodesic Length Morphometry*, the degree of RPI observed in sagittal eye specimens was quantified as arc length and GI, and the degeneration arc length and its location in the entire retina were visualized as the *Geodesic Equidistant-Projection Plot*. The red double arrows in Fig. 6 show the dorsoventral extent of the degeneration. The dash count, *D*, obtained from the specimens in this method is converted into the GI by dividing by *E* and into the actual arc length data by multiplying by *P*.

**Fig. 6:**
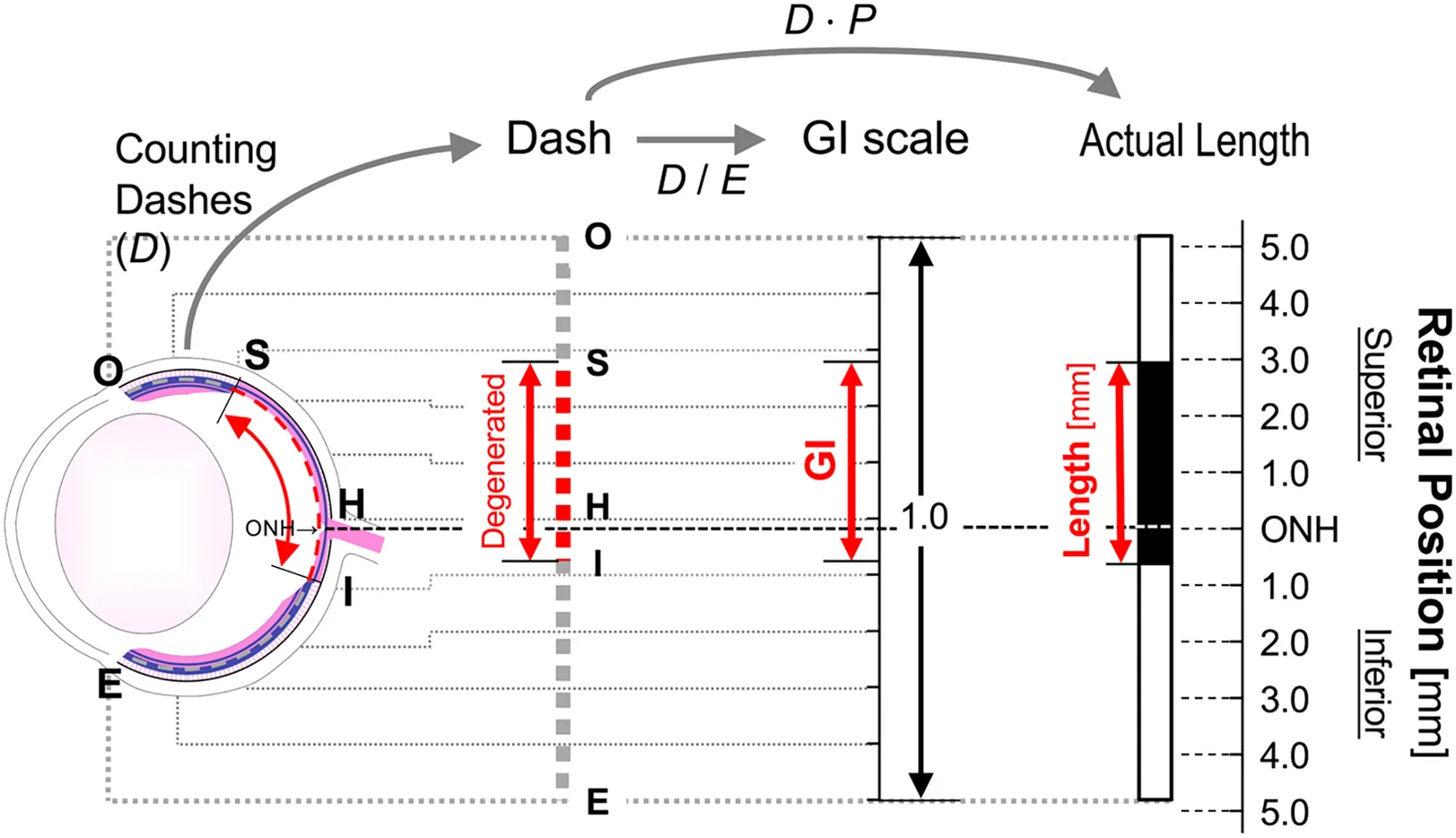
Relationship between the dash count, *Geodesic Index* (GI), and location and arc length of a dorsoventral extent of the retinal degeneration. A summary of the relationship between the pathological image, calculated GI value, and actual arc length. Using dash count, *D*, from O to each position (S, H, I, and E) in the RPI-affected retina, GI and actual arc lengths are calculated. GI is calculated as *D*/*E*, while actual arc length is calculated as *D* ⋅ *P*.

This paper presents a novel method for analyzing retinal images using Microsoft PowerPoint as a general-purpose drawing tool, yielding results comparable to those obtained with dedicated image analysis software such as ImageJ™. By drawing two dashed lines on a retinal image, researchers can obtain positional information on retinal degeneration. A key advantage of this approach is the ability to easily integrate additional information and compile multiple results into a single file, a benefit of using presentation software. Although this method has a minor inherent error because measurements are obtained in dash units, these issues can be mitigated through statistical analysis of multiple specimens. Ultimately, given that both PowerPoint and ImageJ produce similar results, the choice of software and approach can be taken at the researcher's discretion.

This methodology can potentially be applied to studies in other biological fields. The implementation of this methodology requires no specialized equipment beyond standard microscopy, making it highly accessible for any laboratory. Beyond its application to retinal photic injury, this coordinate-based system can be used to map diverse features such as neovascularization, inflammatory cell distribution, or fluorescence distribution profiles. Ultimately, the fundamental principle of projecting morphological changes onto normalized arc length coordinates can be extended to any tissue structures with curved anatomical layers, offering a robust platform for spatial morphometry in various biological fields.

## Method validation

The use of the reference dash pitch, *P*, calibrated against an objective micrometer, ensures the accuracy of the physical scale. This accuracy was further confirmed for curvilinear measurements (Fig. S3), demonstrating that the dash pitch, *P*, is accurately reproduced even in curves.

As a practical application of this validated scale, we determined the pathological data for the rat eye shown in Fig. 3. The measured parameters are as follows: the entire retinal arc length (O-to-E), 10.7 mm; the arc length of the degeneration with no ONL (S-to-I), 6.6 mm; the GI of the degeneration to the entire retina, 0.617; the arc length between the superior ora serrata and the ONH (O-to-H), 5.3 mm; and the arc length between the ONH and the central position of the degeneration (H-to-C), 1.2 mm (Table 1 and Fig. 7).

**Table 1:** Calculation of key parameters and Geodesic Index (GI) for a single retinal photic injury specimen using Geodesic Length Morphometry.

	Range	Dash	Arc length [mm]	GI	Notes
From O to each position	O–S	8	0.8	0.075	
	O–C	41	4.1	0.383	*refer to*Fig. 5
	O–H	53	5.3	0.495	
	O–I	74	7.4	0.692	
Entire retina	O–E	107	10.7	1.000	*refer to*Eq. (1)
Dorsoventral degeneration extent	S–I	66	6.6	0.617	*refer to*Eq. (2)
From ONH to degeneration center	H–C	12	1.2	0.112	*refer to*Eq. (4) and Fig. 5

Raw data, including the retinal arc length, and the corresponding normalized *Geodesic Index* (GI) derived from the histological micrograph of rat RPI shown in Fig. 3. Reference dash pitch, *P*, was used as 0.10 [mm/dash]. Formula and schematic used for arc length calculation are referenced in Methods section.

**Fig. 7:**
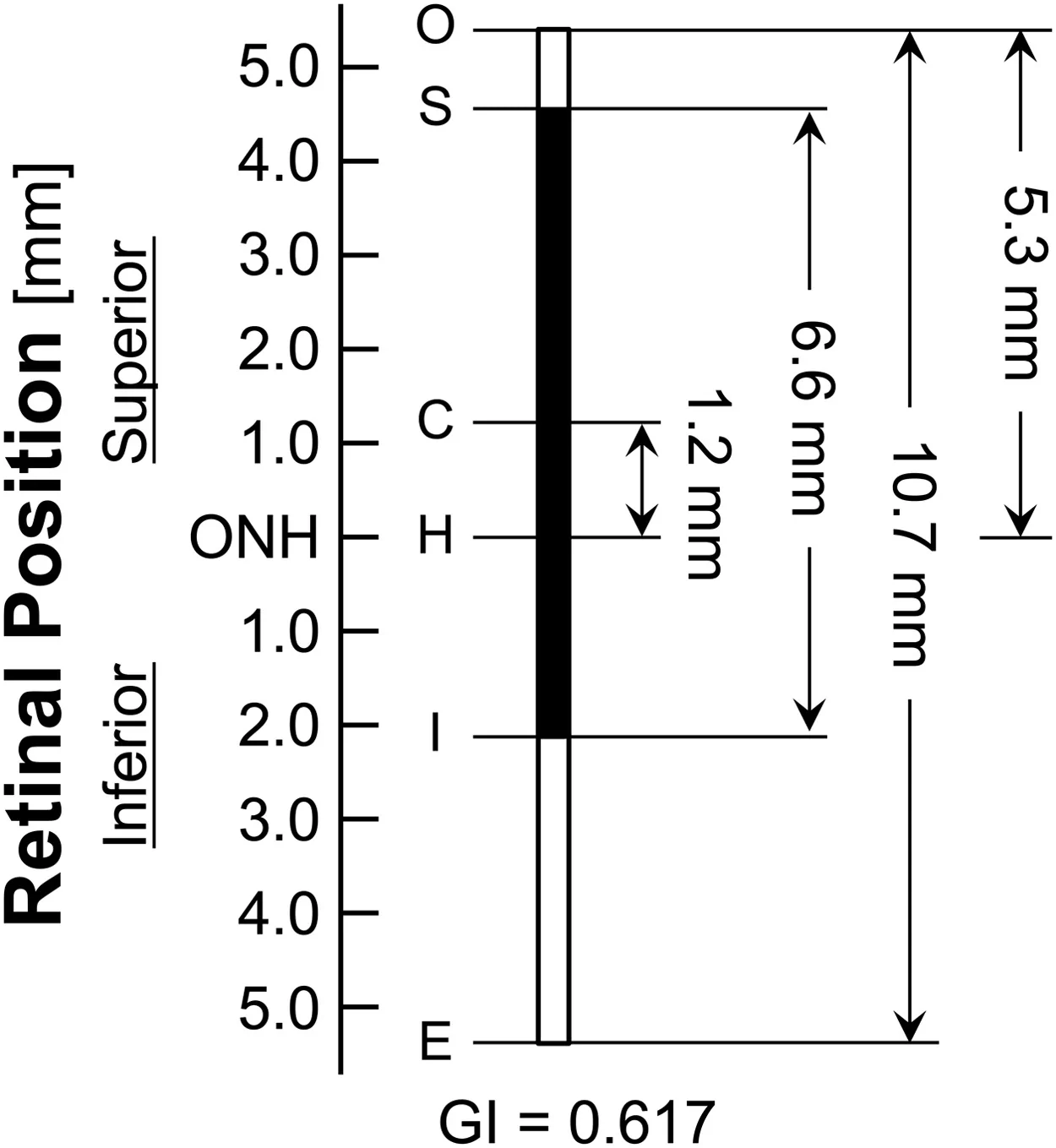
Annotation of morphometric parameters on *Geodesic Equidistant-Projection Plot*. Representative plot (from one eye of a WKY rat) visually demonstrates calculation of major arc length parameters (O–E, O–H, S–I, and C–H) and *Geodesic Index* (GI), corresponding to data shown in Table 1. GI value of the retinal lesion in the eye shown in Fig. 3 is 0.617.

The precision of *Geodesic Length Morphometry* was validated using a cohort of 10 male adult WKY rats (20 eyes), which is representative of highly stable ocular dimensions in a single inbred strain. This validation focused on the precision of the entire retinal arc length and inter-eye differences. The key reliability metrics are summarized in Table 2. The mean entire retinal arc length (O-to-E) was 10.95 ± 0.28 mm [mean ± standard deviation (S.D.)], where the S.D. (0.28 mm) reflects the total biological variability among individuals. The overall coefficient of variation (CV) for this biological variability was 2.52%.

**Table 2:** Reliability of the entire retinal arc length and the inter-eye differences of ten adult male rats using geodesic length morphometry.

	Arc length [mm] (Mean ± S.D.)	Inter-eye difference [mm] (Mean diff. ± S.D.)	Precision (CV)
Entire retinal arc length (O–E)	10.95 ± 0.28	0.27 ± 0.09	0.87%

Values are presented as mean ± S.D. for 10 male adult WKY rats (*n* = 10 [animals]). Arc length mean ± S.D. column shows average dimension and total biological variability among 20 eyes (*N* = 20 [eyes]). Inter-eye difference (mean diff. ± S.D.) column shows average difference between the eyes, where the S.D. (0.09 mm) indicates inherent measurement noise of method. Precision coefficient of variation (CV) of 0.87% is calculated as S.D. of inter-eye difference (0.09 mm) relative to arc length mean (10.95 mm), demonstrating the high quantification precision of the methodology. The raw data for this table are provided in Table S1.

The mean inter-eye difference (mean diff.) was 0.27 ± 0.09 mm, where the S.D. (0.09 mm) effectively isolates and represents the inherent measurement noise of the method. Critically, measurement noise was found to correspond to an exceptionally low precision CV of only 0.87% relative to mean entire retinal arc length (10.95 mm). This CV of 0.87% is significantly lower than the 2.52% biological CV, confirming the high reliability of this method for inter-individual comparison.

This extremely low measurement noise (high stability) can be directly attributed to the methodical design. Furthermore, the method ensures stability and geometric accuracy through the objective definition of anatomical landmarks (O, S, H, I, and E) and the underlying digital processing, which precisely measures curved arc lengths. This consistency confirms the high robustness of this method against typical biological and experimental variations.

### Limitations

As a trade-off for selecting frozen sections in this study—primarily to minimize tissue shrinkage associated with chemical dehydration and embedding—certain technical challenges inherent to this histology occasionally occurred. Specifically, localized tissue irregularities or minor fragmentation can arise due to environmental conditions during sectioning, such as humidity. Despite these minor structural irregularities in some sections, the “*Geodesic Length Morphometry*” approach was still applicable for performing measurements along the retinal contour.

Nevertheless, some inherent limitations remain in this quantification method. First, the initial placement of anchor points used to define the retinal contour relies on the researcher’s subjective visual judgment, introducing a manual element into the tracing process. Second, the spatial resolution of the morphometric scale is determined by the predefined pitch of the dashed line, which is controlled by adjusting the line weight. While a finer pitch achieved through reduced line thickness increases measurement resolution, it also makes manual counting of individual dashes more challenging. Furthermore, since measurements can be significantly affected by the sectioning position within the sample, further optimization of the dash pitch and line weight may be necessary depending on the tissue type and the specific objectives of future studies using this method.

## Ethics statements

All animal experiments were performed in accordance with the ARRIVE guidelines and the National Institutes of Health guide for the care and use of Laboratory animals (NIH Publications No. 8023, revised 1978). This study was approved by the Institutional Animal Care and Use Committee of Hamamatsu University School of Medicine. Furthermore, all procedures were conducted in compliance with the ARVO Statement for the Use of Animals in Ophthalmic and Vision Research. Male (*n* = 10) rats were used in this study.

## CRediT authorship contribution statement

**Kentaro Ohishi:** Conceptualization, Funding acquisition, Methodology, Software, Validation, Visualization, Investigation. **Masafumi Ohtsubo:** Writing – review & editing. **Akira Obana:** Supervision. **Yoshihiro Hotta:** Supervision. **Yu Nagashima:** Writing – review & editing. **Shinsei Minoshima:** Supervision, Writing – review & editing.

## Declaration of interests

The authors declare that they have no known competing financial interests or personal relationships that could have appeared to influence the work reported in this paper.

## Data Availability

The numerical data for 10 male rats are available in Table S1 in the Supplementary Materials.
