# Supplementary material for: GEODESIC LENGTH MORPHOMETRY: A simple method for quantifying severe degeneration laterally in rodent models of retinal photic injury
Source: MethodsX. 2026 Sep 29;17:104183. doi: 10.1016/j.mex.2026.104183 (PMC13694814; doi:10.1016/j.mex.2026.104183)
Supplement: Supplementary file 1 [file mmc1.pdf]

GEODESIC LENGTH MORPHOMETRY: A Simple Method for Quantifying Severe Degeneration Laterally in Rodent Models of Retinal Photic Injury

Kentaro Ohishi,[a,*] Masafumi Ohtsubo,[a] Akira Obana,[a,b] Yoshihiro Hotta,[c] Yu Nagashima,[a]
Shinsei Minoshima [a,d]

[a] Institute of Photonics Medicine, Hamamatsu University School of Medicine, 1-20-1 Handayama, Chuo-ku, Hamamatsu 431-3192, Japan
[b] Department of Ophthalmology, Seirei Hamamatsu General Hospital, 2-12-12 Sumiyoshi, Chuo-ku, Hamamatsu 430-8558, Japan.
[c] Department of Ophthalmology, Hamamatsu University School of Medicine, 1-20-1 Handayama, Chuo-ku, Hamamatsu 431-3192, Japan.
[d] Organization for Innovation and Social Collaboration, Shizuoka University, 3-5-1 Johoku, Chuo-ku, Hamamatsu 432-8561, Japan.

* To whom the correspondence should be addressed
Institute of Photonics Medicine, Hamamatsu University School of Medicine, 1-20-1 Handayama, Chuo-ku, Hamamatsu 431-3192, Japan.
E-mail: kohishi@hama-med.ac.jp
Tel: +81-53-435-2394
Fax: +81-53-435-2394

Notes on Methodology

Software tool selection and practical advantages

For this analysis, we used Microsoft® PowerPoint® for Mac as a general-purpose drawing tool. The "Curve" tool of the software is convenient for drawing a curved line along and through the entire retina with a mouse device. The same measurement can be performed using the "Segmented Line" tool and a combination of the "Fit Spline" and "Measure" commands in ImageJ™ software (NIH, Bethesda, MD) to draw a curve and obtain the arc length. While ImageJ can save these paths in its ROI Manager, reproducing or adjusting multiple partial measurements often requires separate drawing for each segment.

In contrast, the PowerPoint-based method offers several advantages. Unlike ImageJ, in which measurements often require repeatedly drawing of a curve for each desired length, the PowerPoint method requires only a single dashed curvilinear line across the entire retina. This line can be permanently saved in the file, enabling easy and reproducible remeasurements by counting the number of dashes later. Additionally, since the tracing data is directly embedded in the slide and the resulting values can be recorded in the Notes pane, all analytical information is consolidated within a single file. Furthermore, PowerPoint's slide-based format allows numerous analyses to be consolidated into one file by simply adding new slides, which significantly enhances the efficiency of data management and cross-sample analysis. It is important to note, however, that a significant functional limitation exists in this method when using certain older versions of PowerPoint.

Version-dependent inconsistency

For software versions using PowerPoint v2004 for Mac or earlier versions, the relationship between the thick and thin lines is imprecise, and the dashed pitches vary irregularly depending on the slide magnification rate (Fig. S1). Since this method was first developed using PowerPoint v. x, we used the analysis condition with dashed lines of 18 pt and 1.8 pt thickness at a slide magnification level of 110% for several years [dashed line combination with a dash number ratio of 10:1; Fig. S1(C)].

Subsequently, these issues were dramatically improved in PowerPoint v2008 for Mac and completely resolved by v2019 (Fig. S1), providing greater flexibility in defining the dashed pitches, which directly determine the measurement resolution. This improvement in drawing precision reflects the evolution of the Mac graphics stack as a rendering engine:

QuickDraw (–v2004)

↓

Core Graphics/Quartz 2D (v2008–v2011)

↓

Quartz 2D + Metal GPU acceleration (v2016+)

Architectural shift and mathematical verification of drawing precision

The transition from legacy binary formats (. ppt) to an eXtensible Markup Language (XML)-based format (. pptx), which was initiated by the release of PowerPoint v2008 for Mac, brought about a fundamental shift in the definition and storage of graphical information. XML provides explicit, absolute, and vector definitions. As graphical objects are represented as structured text data, their geometric properties can be explicitly and numerically defined using coordinate values. This architectural transition ensures consistent and scale-independent rendering of dashed lines, which is an essential factor for the present analytical method, thereby ensuring the integrity of the coordinate data across variations in line thickness, resizing, or slide scaling.

In this study, the involvement of the Catmull–Rom spline algorithm was verified by extracting the anchor-point coordinates directly from the Office Open XML data. By substituting these coordinates into the Catmull–Rom spline formulas (shown below), the calculated first and second control points were found to be identical to those stored within the cubic Bézier (“cubicBezTo”) elements. This confirms the mathematical validity of the tracing process using the “Curve” tool in PowerPoint.

For a segment between anchor points P_i and P_{i+1}, the control points C_1 and C_2 in the Catmull–Rom spline curves are defined as:

Start point (P_i): P_i

First control point (C_1): $$C_1 = P_i + \frac{P_{i+1} - P_{i-1}}{6}$$

Second control point (C_2): $$C_2 = P_{i+1} - \frac{P_{i+2} - P_i}{6}$$

End point (P_{i+1}): P_{i+1}

The anchor and control points are plotted on a cubic Bézier curve, as shown in Fig. S2.

Validation of geometric accuracy of Geodesic Length Morphometry

The fundamental assumption of *Geodesic Length Morphometry* is that the reference dash pitch P remains consistent regardless of the curvature of the measured path. To validate this geometric accuracy, five concentric dashed circles (A–E) with decreasing circumference were generated in Microsoft PowerPoint for Mac using the same dash thickness settings (1.35 pt and 13.5 pt) as the actual measurement (Fig. S3). The actual circumferences of these circles were calculated from their precise diameters. The dashed lines were drawn along the circumference of each circle, and the number of dashed segments counted for the entire circumference was recorded. The consistency of the ratio of the actual circumference to the counted dashed segments across the five curved paths (A–E) confirmed that the measurement results of *Geodesic Length Morphometry* are linearly scaled with the actual arc length and are not influenced by the

degree of curvature. This validation establishes the reliability of using the *P* value for the accurate quantification of curved anatomical structures such as the retinal arc length.

Prerequisites and specific parameters for Geodesic Length Morphometry

The *Geodesic Length Morphometry* requires the following three criteria to be met:

1. Selection of a distinct pathological feature of interest suitable for quantification (this feature can encompass findings from standard histology, *in situ* hybridization, or immunohistochemical staining).
2. Definition of extractable positions (*e.g.*, O, S, H, I, and E) and corresponding variables (*e.g.*, *S*, *H*, *I*, and *E*) of the outer retina;
3. Drawing a dashed curve that accurately reflects the overall arc length of the retina.

To analyze the mouse eye, a suitable objective lens magnification of 4× (rat eye: 2×) was used, and the *P* value should be calculated with a stage micrometer microgram taken with a 4× objective lens.

Consistency across different measurement parameters

It is important to note that the primary measurements presented in this study used the pitch condition of $P = 0.10$ mm/dash (Fig. 1), which was determined using the latest consistent method. However, most previous measurements by our group utilized an older parameter set ($P \approx 0.133$ mm/dash), as detailed in Fig. S4. While the transition to the $P = 0.10$ mm/dash parameter improved the precision of the absolute measurements by enhancing the spatial sampling density, the core biological conclusions remained consistent with those obtained using the previous $P \approx 0.133$ mm/dash setting. This demonstrates that the *Geodesic Length Morphometry* method is highly robust; while a finer pitch provides superior resolution, the relative biological trends are preserved across different parameter sets.

Reference

[10] K. Ohishi, M. Ohtsubo, A. Obana, Y. Hotta, Y. Nagashima, S. Minoshima, Morphometric analysis of retinal photic injury susceptibility in rats: A triphasic severity classification based on post-injury phenotypes, *Exp. Eye Res.*, **272** (2026) 111166. doi: 10.1016/j.exer.2026.111166.

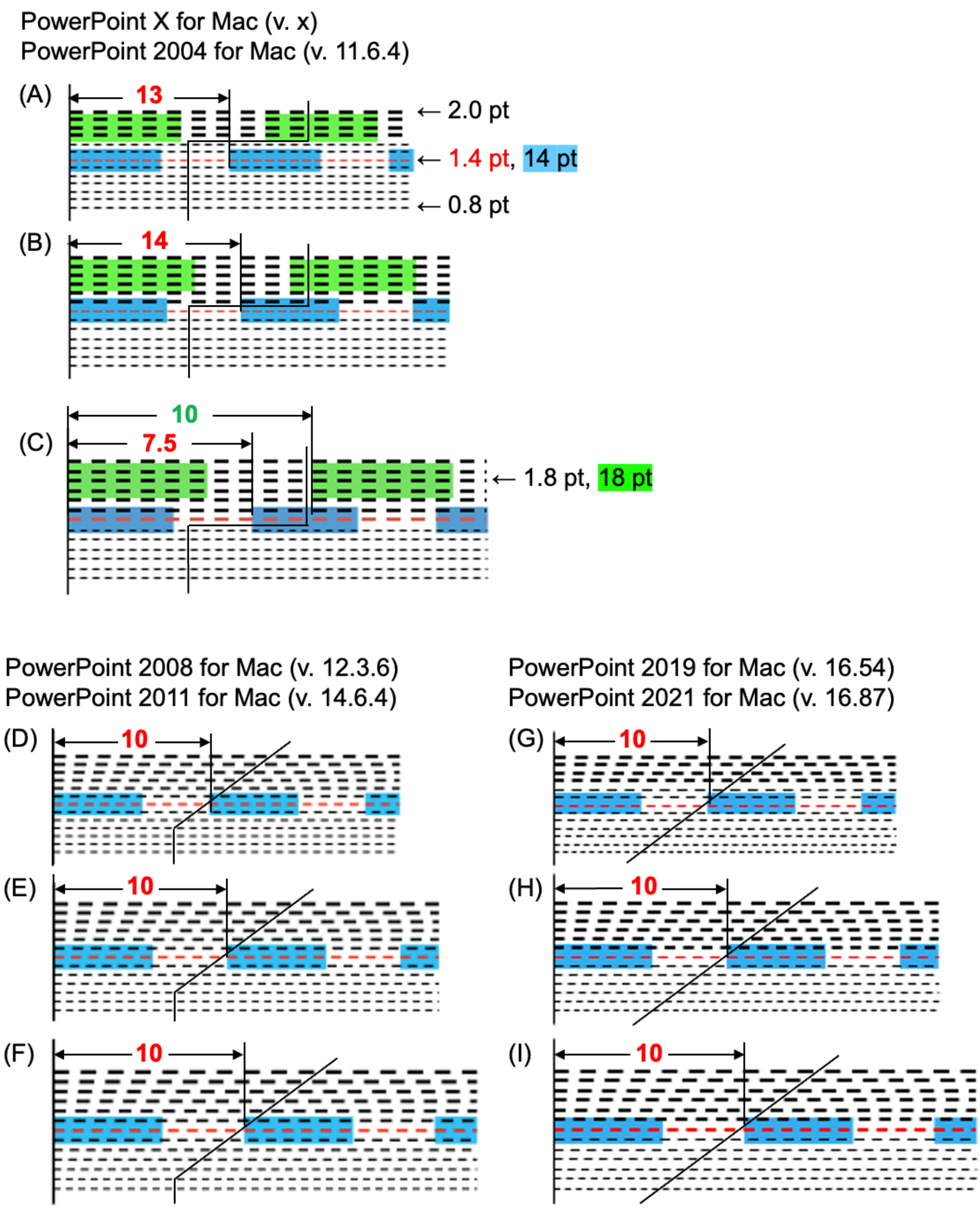

Fig. S1. Relationship between a dash pitch and a slide expansion rate in different software versions.

PowerPoint for Mac version: (A-C) vX/v2004, (D-F) v2008/v2011, (G-I) v2019/v2021 (the results for each group are the same in both versions); slide's magnification levels [percentage values specified in the percent edit box (available in the "Zoom" group on the "View" tab)]: (A, D, G) 90%, (B, E, H) 100%, and (C, F, I) 110%; thickness settings for the thin dashed lines: 0.8 pt to 2.0 pt in 0.1 increments from the bottom; thick dashed line (blue): 14 pt thickness; the thin dashed line (red): 1.4 pt thickness. The thick dashed green lines (A-C) are 18 pt in thickness. The hooked and diagonal lines indicate the 10th dash reference position for each thin dashed line. These lines were used to visually confirm the consistency of the relative positions of the dashed lines across slide magnification levels and software versions.

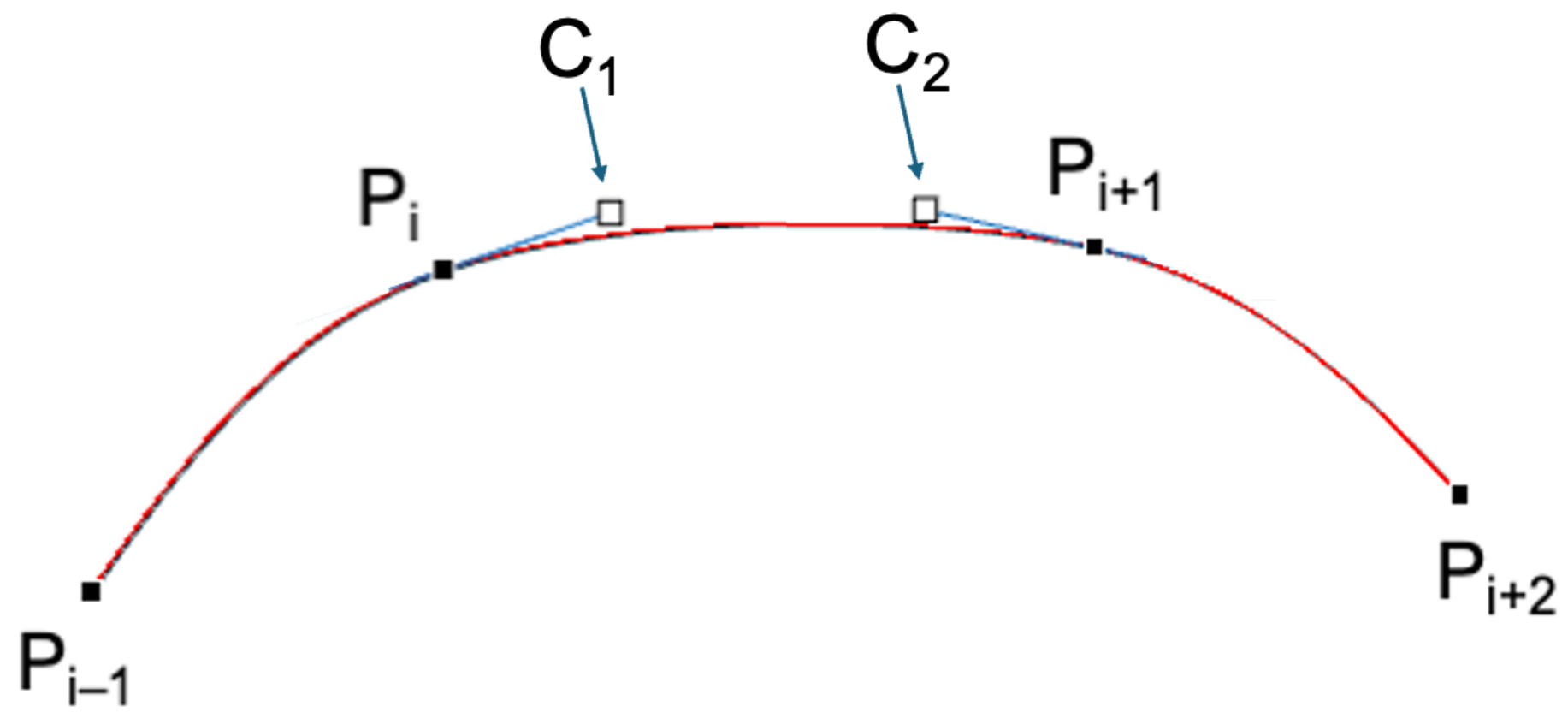

Fig. S2. **An example of a cubic Bézier curve with the first and second control points (C_1 and C_2, respectively) distributed conforming to the Catmull–Rom spline algorithm.**

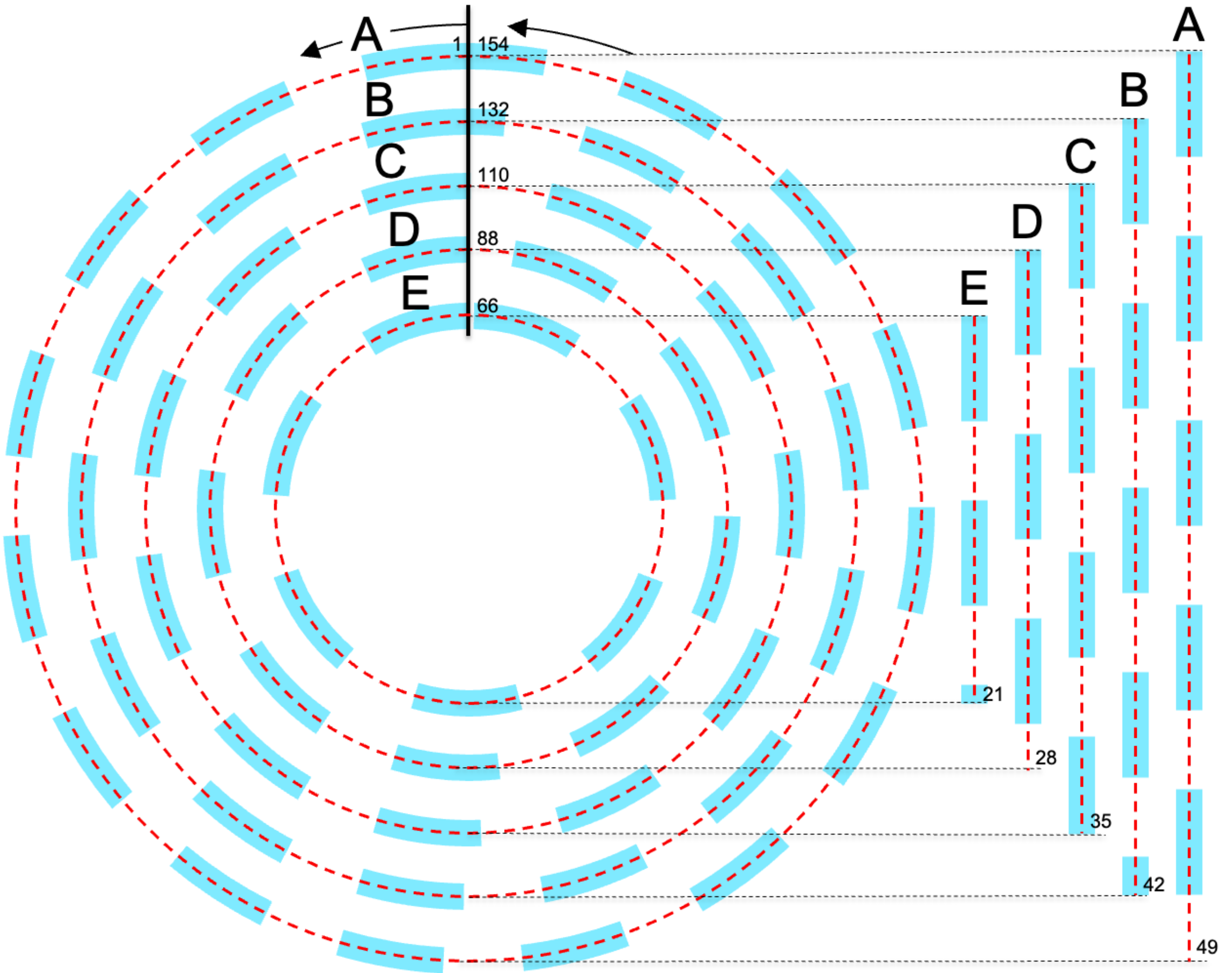

Fig. S3. Validation of the geometric accuracy of the Geodesic Length Morphometry using dashed circles.

Geometric accuracy of the reference dash pitch, *P*, in curvilinear measurements was verified using five concentric dashed circles with decreasing circumferences and their corresponding diameter lines (A-E) with thicknesses of 1.35 pt (red) and 13.5 pt (blue). Counted dash segments for the circumference were compared against calculated diameter (Circumference / π) to confirm consistency of *P* across curved paths.

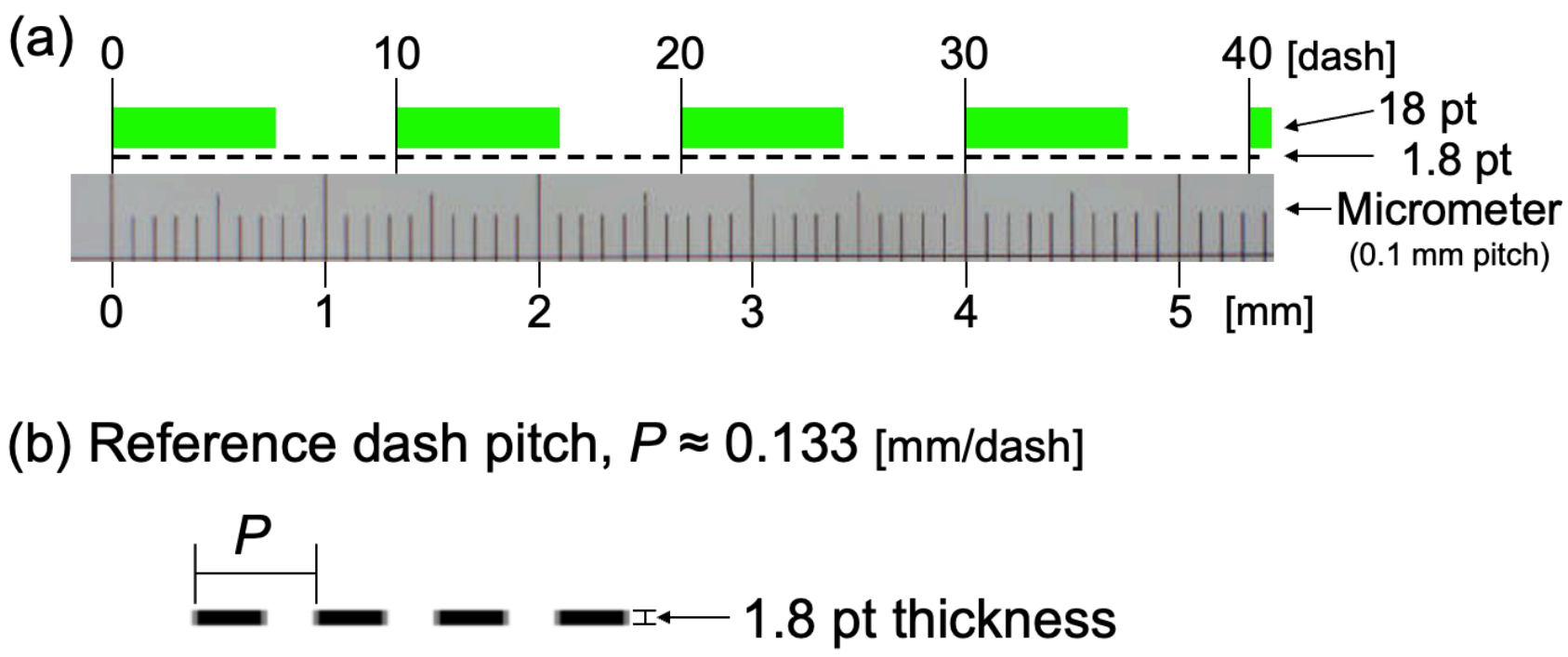

Fig. S4. Determination of the reference dash pitch using a stage micrometer micrograph.

(a) Thin (1.8 pt) and thick (18 pt) dashed lines were drawn next to stage micrometer micrograph. Numbers above dashed lines indicate number of dashes from left edge position in thin dashed line. Dash number ratio between thin and thick dashed lines is 10:1. (b) A reference dash pitch, *P*, is calculated to be *ca.* 0.133 mm/dash, since the 30th-dash position on 1.8-pt dashed line corresponds to 4.0 mm in (a).

Table S1. Raw data of retinal arc lengths in both eyes of 10 male WKY rats.

	Strain	ID No.	Sex	Age of Sacrifice (week)	Dash number		Retinal arc length (mm)		
					1st eye	2nd eye	1st eye	2nd eye	Diff.
1	WKY	979	♂	26	111	109	11.1	10.9	0.2
2	WKY	991	♂	38	113	117	11.3	11.7	0.4
3	WKY	978	♂	26	106	105	10.6	10.5	0.1
4	WKY	993	♂	38	110	108	11.0	10.8	0.2
5	WKY	977	♂	26	106	110	10.6	11.0	0.4
6	WKY	976	♂	26	110	107	11.0	10.7	0.3
7	WKY	975	♂	26	110	107	11.0	10.7	0.3
8	WKY	973	♂	28	112	109	11.2	10.9	0.3
9	WKY	971	♂	28	108	111	10.8	11.1	0.3
10	WKY	970	♂	28	111	109	11.1	10.9	0.2

Note: Strain, rat ID, sex, weeks, and age at sacrifice are sourced from our previous study [10]. The dash numbers and retinal arc lengths represent new morphometric data obtained in the present study.
